# HIV-1 Protease Inhibitors Slow HPV16-Driven Cell Proliferation through Targeted Depletion of Viral E6 and E7 Oncoproteins

**DOI:** 10.3390/cancers13050949

**Published:** 2021-02-24

**Authors:** Soyeong Park, Andrew Auyeung, Denis L. Lee, Paul F. Lambert, Evie H. Carchman, Nathan M. Sherer

**Affiliations:** 1 McArdle Laboratory for Cancer Research, Deptartment of Oncology, University of Wisconsin School of Medicine and Public Health, Madison, WI 53705, USA; spark64@wisc.edu (S.P.); denis.lee@wisc.edu (D.L.L.); plambert@wisc.edu (P.F.L.); 2 Institute for Molecular Virology, University of Wisconsin-Madison, Madison, WI 53706, USA; 3 Carbone Cancer Center, University of Wisconsin School of Medicine and Public Health, Madison, WI 53705, USA; vndrem48620@gmail.com (A.A.); carchman@surgery.wisc.edu (E.H.C.); 4 Department of Surgery, University of Wisconsin School of Medicine and Public Health, Madison, WI 53792, USA

**Keywords:** HPV, protease inhibitors, E6, E7, cervical cancer

## Abstract

**Simple Summary:**

High-risk strains of human papillomavirus (HPV) such as HPV16 cause anogenital and oropharyngeal cancers, with HPV-associated cervical cancer being the fourth most common cancer worldwide and a major cause of death for women. Anti-HPV vaccines are available but coverage is low, and while the vaccines prevent infection they are not therapeutic for treating cancers in individuals already infected with high-risk HPV. Instead, current treatments for HPV-associated cancers include surgery, chemotherapy, and radiation, and affect diseased and healthy tissues alike. Accordingly, there is a dire need for better, molecularly targeted strategies. Herein, we report the novel finding that at least four protease inhibitor drugs currently used to treat HIV/AIDS—lopinavir, ritonavir, nelfinavir, and saquinavir—cause marked and selective depletion of HPV16 E6 and E7 oncoproteins in HPV-positive cancer cells and a 3-D cell culture model for HPV16-positive epithelial pre-cancer (NIKS16 cells). E6 and E7 depletion correlated with anticancer phenotypes including increased p53 protein levels and induction of cell growth arrest in CaSki cells as well as slowed epithelium growth and reversal of HPV16-driven phenotypes consistent with inhibition of the cellular autophagy pathway in the NIKS16 model. Based on this progress in vitro, we propose that HIV protease inhibitors, already known to have anticancer properties, be studied further in the context of developing a targeted therapy for HPV16-driven cancers and pre-cancers.

**Abstract:**

High-risk human papillomavirus strain 16 (HPV16) causes oral and anogenital cancers through the activities of two viral oncoproteins, E6 and E7, that dysregulate the host p53 and pRb tumor suppressor pathways, respectively. The maintenance of HPV16-positive cancers requires constitutive expression of E6 and E7. Therefore, inactivating these proteins could provide the basis for an anticancer therapy. Herein we demonstrate that a subset of aspartyl protease inhibitor drugs currently used to treat HIV/AIDS cause marked reductions in HPV16 E6 and E7 protein levels using two independent cell culture models: HPV16-transformed CaSki cervical cancer cells and NIKS16 organotypic raft cultures (a 3-D HPV16-positive model of epithelial pre-cancer). Treatment of CaSki cells with some (lopinavir, ritonavir, nelfinavir, and saquinavir) but not other (indinavir and atazanavir) protease inhibitors reduced E6 and E7 protein levels, correlating with increased p53 protein levels and decreased cell viability. Long-term (>7 day) treatment of HPV16-positive NIKS16 raft cultures with saquinavir caused epithelial atrophy with no discernible effects on HPV-negative rafts, demonstrating selectivity. Saquinavir also reduced HPV16′s effects on markers of the cellular autophagy pathway in NIKS16 rafts, a hallmark of HPV-driven pre-cancers. Taken together, these data suggest HIV-1 protease inhibitors be studied further in the context of treating or preventing HPV16-positive cancers.

## 1. Introduction

Human papillomaviruses (HPVs) are non-enveloped DNA tumor viruses that infect stratified squamous epithelial tissues. High-risk mucosotropic strains cause over 99% of cervical cancers, most other epithelial anogenital cancers, and a subset of head and neck cancers. Safe and effective, multi-valent anti-HPV vaccines are available [1,2]. However, anti-HPV vaccines are non-therapeutic and coverage remains low; e.g., only 68% of adolescents in the U.S. received the first of two prescribed doses in 2018 [3]. For cervical cancer, nearly 200,000 pre-cancer cases are still diagnosed each year, with ~11,000 cases of full-blown cancer causing ~4000 deaths annually [4]. Current approaches to treat HPV-associated cancers include surgery, chemotherapy, and radiotherapy, with pre-cancerous cervical lesions treated using ablative procedures such as the Loop Electrosurgical Excision Procedure. All are poorly efficacious, non-targeted, and can cause side effects and loss to quality of life [5]. Accordingly, there is a strong need to identify approaches for targeted, less invasive therapies, in particular to treat HPV-positive pre-cancers in the context of cancer prevention.

HPV causes cancer through the activities of three viral oncoproteins: E5, E6, and E7. Whereas E5′s importance varies depending on virus strain and cancer, HPV E6 and E7 are expressed in all HPV-positive cancers [6,7]. E6 and E7 promote cancer development through synergistic mechanisms, manipulating host cellular pathways that drive the cell cycle, cause genomic instability, and promote immune evasion [6,7]. The most well-known targets of E6 and E7 are p53 and pRb, respectively. E6 suppresses the p53 tumor suppressor pathway by recruiting E6-associated protein (E6-AP), an E3 ubiquitin ligase that ubiquitinates p53 and marks it for proteasome-mediated degradation [8,9,10,11]. E6 has also been shown to be capable of activating host telomerase and/or dysregulating the function of PDZ proteins through separate mechanisms, with both targets thought to promote viral replication while increasing the risk of cell transformation [12,13,14,15,16,17,18]. E7 inactivates pRb “pocket” family proteins that typically function to regulate cell cycle progression [19,20]. E7 can also inhibit pRb-activating proteins including cyclin-dependent kinase inhibitors p21^CIP1^ and p27^KIP1^ [21]. 

During productive HPV infection, E6 and E7 expression is tightly controlled to play critical, temporally regulated roles in reprogramming infected epithelial cells to support viral replication [22]. Viral persistence in the epithelium is a prerequisite for developing HPV-positive cancers, with cancer progression typically initiated when portions of the HPV dsDNA genome become integrated into the host cell DNA causing dysregulated, constitutive expression of E6 and E7 [23,24]. Continued expression of E6 and E7, virally induced epigenetic dysregulation of host gene expression, and subsequent acquisition of mutations in cancer-related host genes (e.g., gain of function mutations in *PI3KCA* or loss of function mutations in *Notch*) all contribute to malignant progression, a process that typically takes many years from the time of infection to development of HPV-driven pre-cancer [15,25].

People living with human immunodeficiency virus type 1 (HIV-1) infection (PLWH) are at elevated risk for developing HPV-associated cancers, with this risk only partially mitigated by highly active antiretroviral therapy (HAART) [26,27]. HAART involves oral administration of a cocktail of anti-HIV-1 drugs that include two nucleos(t)ide reverse transcriptase inhibitors (NRTIs) and either a non-nucleoside RT inhibitor (NNRTI), integrase inhibitor (INSTI), or protease inhibitor (PI) [28]. HIV-1 protease inhibitors block HIV-1′s aspartyl protease (encoded in the viral Gag-Pol protein) in order to prevent viral capsid maturation and thus abolish viral infectivity. Although these drugs are potent, standard HAART therapy today routinely excludes protease inhibitors due to off-target effects during long-term treatment causing conditions that include glucose intolerance, lipohypertrophy, and/or other lipid disorders. Interestingly, protease inhibitors, in particular nelfinavir, have also been studied independently of HIV-1 as anticancer agents based on their capacity to trigger cancer cell death, thought to be mediated through inhibition of the host ubiquitin-proteasome system (UBS) [29,30,31]. These benefits have previously been demonstrated in the context of HPV-positive cancers [32,33,34,35] including clinical data from Hampson and colleagues demonstrating that a combination of HIV protease inhibitors (lopinavir and ritonavir) causes regression of cervical dysplasia in HPV-positive women with high-grade cervical intraepithelial neoplasia [36]. 

Here, the complexity of HIV-HPV interactions and persistence of HPV disease in PLWH, combined with the fact that certain protease inhibitors have proven effective in treating HPV-positive cervical cancer and its precursor lesions [32,33,34,35,36], prompted us to study the specific effects of protease inhibitors on HPV16 E6 and E7 gene expression using two HPV-positive cell culture models: (1) cervical cancer-derived CaSki cells carrying more than 200 integrated copies of the high-risk HPV16 genome, and (2) normal immortalized human foreskin keratinocytes harboring extrachromosomal HPV16 DNA (NIKS16 cells) that grow in organotypic (raft) culture to form a stratified, 3-D biomimetic model of HPV-positive epithelial pre-cancer. We report the surprising finding that a subset of PIs (lopinavir, rotinavir, nelfinavir, and saquinavir) markedly reduce levels of HPV16 E6 and E7 proteins in both CaSki and NIKS16 cells correlating with suppression of HPV16-positive cell growth. The effects occurred downstream of E6 and E7 transcription and were at least partially reversed by a proteasome inhibitor (MG132), suggesting that protease inhibitors cause depletion of E6 and E7 by triggering protein destabilization. Protease inhibitors were also found to reverse HPV16-driven increases to markers of cellular autophagy in NIKS16 rafts, a potentially significant result because induction of autophagy has also been shown to reduce HPV’s oncogenic potential [37]. These results provide additional insights into how HIV protease inhibitors interfere with HPV-associated carcinogenesis, and further support the notion that these drugs be studied further in the context of targeted treatment or prevention of HPV-driven cancers.

## 2. Results

### 2.1. A Subset of HIV-1 Protease Inhibitors Reduce Levels of HPV16 E6 and E7 Oncoproteins, Correlating with Increased Levels of p53 and HPV-Positive Cancer Cell Death

**Figure 1:**
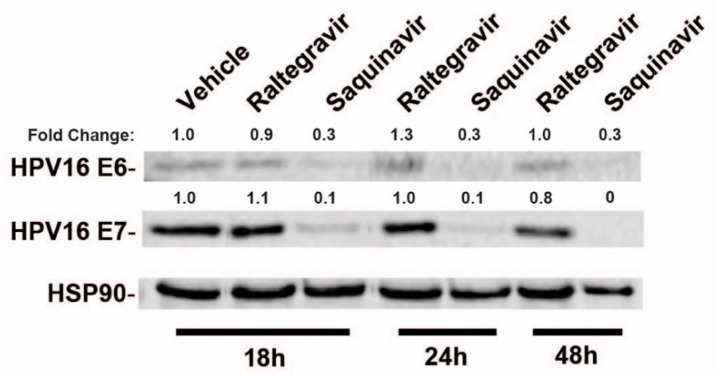
HPV16-positive CaSki cervical cancer cells were incubated in the presence of vehicle (DMSO), 2 µM raltegravir (HIV-1 integrase inhibitor) or 5 µM saquinavir (HIV-1 protease inhibitor) for the indicated time intervals prior to cell harvest and immunoblot using anti-HPV16 E6, anti-HPV16 E7, and anti-HSP90 (control) primary antisera. Numbers are based on infrared fluorescence detection with levels normalized to vehicle (DMSO) control.

To test the effects of a subset of commonly used HIV-1 drugs on HPV16 gene expression, we first compared the effects of raltegravir (RGV), an HIV-1 integrase inhibitor, to saquinavir (SQV), an HIV-1 protease inhibitor (PI), on HPV16 E6 and E7 gene expression in HPV16+ CaSki cells. CaSki cells were treated with HIV-ablative concentrations [38,39,40] of RGV or SQV for 18, 24, or 48 h with cell lysates harvested and HPV16 E6 and E7 levels measured using quantitative immunoblot. RGV had no effect on E6 and E7 levels but SQV caused a marked decline in both E6 and E7 protein levels over time (Figure 1).

**Figure 2:**
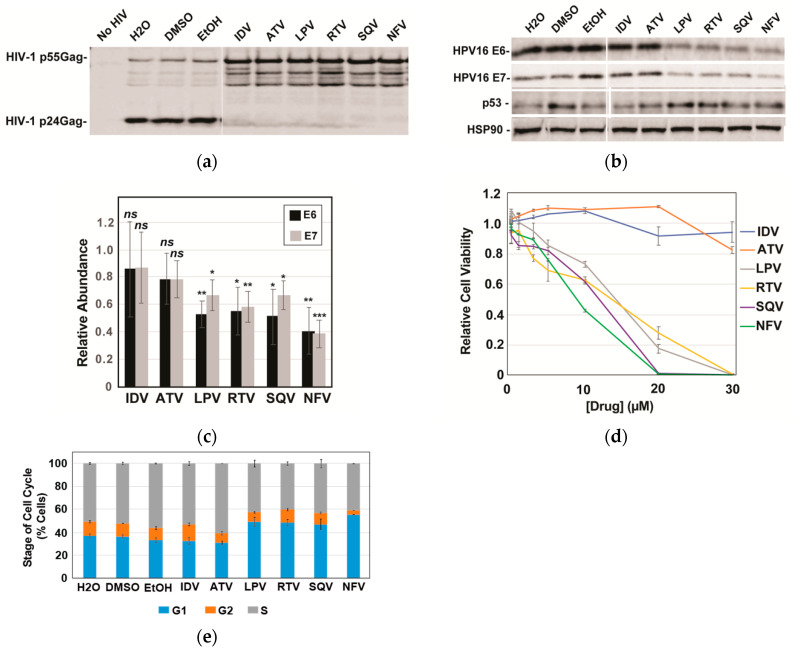
A subset of HIV-1 protease inhibitors reduces HPV16 E6 and E7 expression, correlating with increases to p53 and reductions to cell viability. HPV16-transformed CaSki cells were incubated in the presence of vehicle (H_2_O for ADV and ATV; DMSO for LPV, RTV, and SQV; and EtOH for NFV) or 10 µM of each indicated PI for 48 h prior to analysis. (**a**) HIV-1 particles were harvested from infected CaSki cell supernatants at 48 h post-infection, sedimented, and analyzed for inhibition of HIV-1 protease activity based on loss of viral Gag/Gag-Pol cleavage using immunoblot and anti-p24^Gag^ antiserum. (**b**) In a parallel experiment, CaSki cells were lysed with E6, E7, and HSP90 (control) detected as for Figure 1 with p53 detected using anti-p53 antiserum. Immunoblots are representative of three independent experiments. (**c**) Quantification of E6 and E7 levels in CaSki cells based on quantitative immunoblot as for 2B with averages relative to vehicle control determined after performing three biological experimental replicates. Error bars represent standard deviation of the mean. ns = not significant; * = *p* ≤ 0.05; ** = *p* ≤ 0.005; *** = *p* ≤ 0.0005 based on a Student’s two-tailed *t*-test. (**d**) Cell viability relative to vehicle control measured at 72 h post-treatment for CaSki cells incubated with each of the six drugs at the indicated concentrations. Experiment was carried out in technical triplicate with error bars representing standard deviation of the mean. (**e**) Effects of protease inhibitors (10 µM) on the cell cycle at 72 h post-treatment determined using DAPI labeling and flow cytometry as described in materials and methods. G1, G2, and S phase distributions were plotted as percent of cell population with error bars representing the standard deviation of the mean for each individual stage. Data derived from three technical experimental replicates.

Based on the striking effects of SQV on E6 and E7, we subsequently compared SQV to five additional FDA-approved protease inhibitors: indinavir (IDV), atazanavir (ATV), lopinavir (LPV), ritonavir (RTV), and nelfinavir (NFV). Protease inhibitor activity was first confirmed using an HIV virion cleavage assay wherein CaSki cells were transduced using a single-round HIV-1 reporter virus and then treated with drug (10 µM) for 48 h prior to scoring for HIV-1 Protease-mediated Gag/Gag-Pol processing in harvested virus-like particles. All six protease inhibitors inhibited Gag/Gag-Pol cleavage to a similar extent (Figure 2a). Interestingly, however, of the six protease inhibitors tested only LPV, RTV, SQV, and NFV caused significant reductions to HPV16 E6 and E7 levels, with NFV yielding the strongest effects relative to vehicle control (Figure 2b with quantification in Figure 2c). Because E6 causes ubiquitin-mediated degradation of p53 [9] and because LPV had previously been shown to increase levels of p53 in E6-expressing cancer cells [34], we also monitored p53 protein levels in this experiment, observing moderate increases to p53 for the four protease inhibitors that decreased levels of E6 and E7 (Figure 2c). These results implicated some (LPV, NFV, RTV, SQV) but not all (ATV and IDV) HIV-1 protease inhibitors as inhibitors of E6 and E7 expression in CaSki cells, with the drugs’ converse effects on p53 showing that the mechanism was selective, i.e., not reflecting global suppression of cellular gene expression.

**Figure 3:**
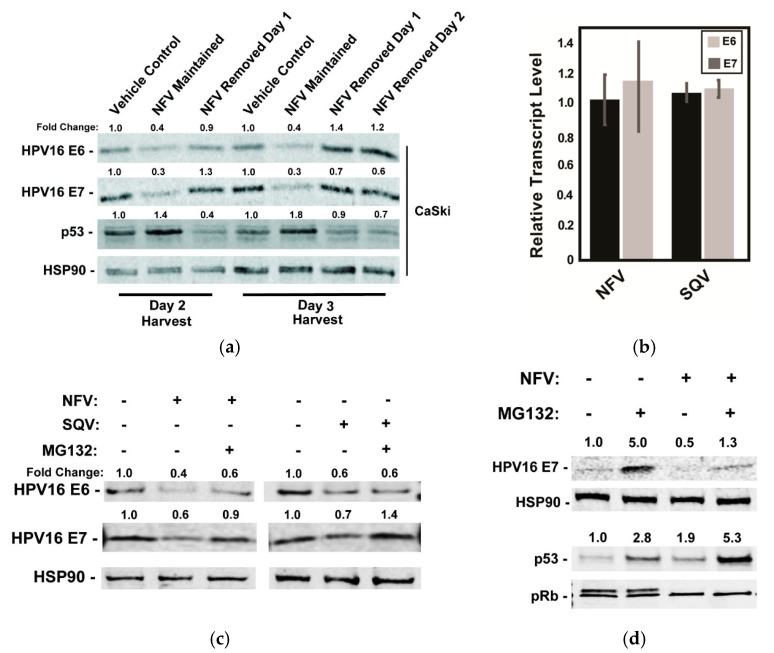
Effects of HIV-1 protease inhibitors on E6 and E7 protein levels are reversible and operate after *E6* and *E7* mRNA transcription. (**a**) CaSki cells were treated with 10 µM NFV for 24 h to 72 h with the drug either maintained or removed as indicated. Cell lysates were harvested at day 2 or day 3 and probed for HPV16 E6, HPV E7, p53, and HSP90 (control) protein levels using quantitative immunoblot, with values normalized to the vehicle control. Data are representative of two biological replicates. (**b**) Levels of *E6* and *E7* mRNA relative to *GAPDH* control in CaSki cells measured 48 h after addition of NFV or SQV using qRT-PCR, with data further normalized to vehicle-treated control cells. Error bars represent the standard deviation of the mean for three technical experimental replicates. (**c**) Effects of NFV and SQV on HPV16 E6 and E7 levels in the presence or absence of the proteasome inhibitor MG132. CaSki cells were treated with 10 µM NFV or SQV for 48 h prior to the addition of vehicle (DMSO) or 5 µM MG132 for 3 h and harvesting lysates for quantitative immunoblot. HSP90 served as a loading and expression control. Relative values were normalized to the vehicle control. Data are representative of three biological replicates. (**d**) As with (**c**), HPV16 E7, HSP90, p53, and pRb were measured by immunoblot after NFV treatment in the presence or absence of MG132. Data are representative of three biological replicates.

Gene silencing of HPV16 E6 and/or E7 has previously been shown to slow the growth of HPV16-positive cervical cancer cells both in vitro and in vivo [41,42,43,44,45], prompting us to next evaluate the effects of HIV-1 protease inhibitors on CaSki cell viability and the cell cycle (Figure 2d,e). As anticipated, the four protease inhibitors (LPV, RTV, SQV, and NFV) that caused reductions to E6 and E7 levels and increased p53 levels caused enhanced cell death over 72 h in CaSki cells relative to IDV and ATV (Figure 3d, left panel), measured using the CellTiter-Glo Assay (Promega). Cell cycle analysis using DNA staining and flow cytometry indicated that these effects were at least in part due to increased numbers of cells accumulating in the G1 phase of the cell cycle (Figure 2e).

### 2.2. The Effects of Protease Inhibitors on HPV16 E6 and E7 Proteins in CaSki Cells Are Reversible and Operate Post-Transcriptionally

To begin to address the mechanism by which the protease inhibitors caused decreases in levels of the HPV oncoproteins, we tested the durability of protease inhibitor effects on E6 and E7 levels in CaSki cells by adding NFV and then either maintaining or washing out the drug at 24 or 48 h post-treatment prior to 24 h further incubation in the absence of drug and cell harvest (Figure 3a). As expected, 10 µM NFV caused measurable decreases to E6 and E7, correlating with increases to p53 levels detected as early as 48 h post-treatment (Figure 3a). After drug removal, however, levels of E6, E7, and p53 proteins returned to levels seen in untreated CaSki cells within 24 h, demonstrating that NFV’s effects are reversible (Figure 3a). This result ruled out long-term epigenetic suppression (e.g., promoter silencing) and was more consistent with transient suppression of E6/E7 transcription, translation or the drugs having post-translational effects (e.g., increasing rates of protein turnover).

We found suppression at the transcriptional level to be the most attractive hypothesis because E6 and E7 share a common promoter [46] and lack protein sequence similarity [47,48]. Moreover, SQV and NFV were previously shown to exert global inhibition of host proteasomes [49], which would be a mechanism hard to reconcile with the observed decrease to viral oncoprotein levels over time. Contrary to this hypothesis, however, 48h of NFV or SQV treatment had virtually no effect on E6 and E7 mRNA levels when compared to untreated cells using qRT-PCR (Figure 3b). By contrast, the addition of the proteasome inhibitor MG132 for a short period of 3 h, starting at 48 h post-treatment with NFV or SQV, caused moderate to full recovery of each viral oncoprotein (Figure 3c). Although the effects of MG132 were variable, these results were more consistent with an alternative hypothesis that protease inhibitors affect E6 and E7 at the level of translation or protein turnover. 

In a parallel experiment, we assayed the effects of NFV on E7, HSP90, p53, or pRb with or without addition of MG132, observing that MG132 only partially rescued E7 levels in the presence of NFV, and that the combined effects of NFV and MG132 on p53 were additive (Figure 3d). The p53 result was again in agreement with NFV affecting p53 due to reduced E6 activity, as opposed to a more general effects on host proteasome function. We also observed that, while NFV had no major effects on pRb protein level, it caused the striking loss of a second, more slowly migrating pRb band detected in immunoblots (Figure 3d). A similar phenotype was observed in a previous study after shRNA-mediated silencing of E7 expression, with the loss of the pRb doublet shown to be due to pRb hypophosphorylation [50]. That pRb hypophosphorylation is known to occur during the G1 phase of the cell cycle [51] may indicate that the accumulation of cells in G1 we observed after treatment with protease inhibitors (Figure 2e) was a direct consequence of reduced E7 levels. Taken together, both of these host cell responses, affecting p53 and pRb after protease inhibitor treatment, were consistent with anticancer phenotypes expected to occur in response to E6 and E7 inactivation.

### 2.3. Saquinavir Slows Cell Growth and Reverses HPV16-Associated Increases to Markers of Autophagy in HPV16-Positive NIKS16 3-D Organotypic Raft Cultures

Because CaSki cells are HPV16-positive cancer cells carrying integrated HPV16 genomes, we next wanted to test the effects of protease inhibitors in a model of pre-cancerous HPV16 infection: normal immortalized keratinocytes (NIKS, originally called BC-1-Ep/SL cells) engineered to carry 10–50 extrachromosomal HPV16 genomes per cell (NIKS16 cells) [52]. NIKS are a spontaneously immortalized keratinocyte cell line (NIKS) derived from human foreskin [53]. When grown on collagen in 3D organotypic raft culture, NIKS form a biomimetic epithelium similar to human skin; NIKS16 cells support the entire HPV16 replication cycle starting in basal cells and progressing over the course of epithelial cell differentiation.

**Figure 4:**
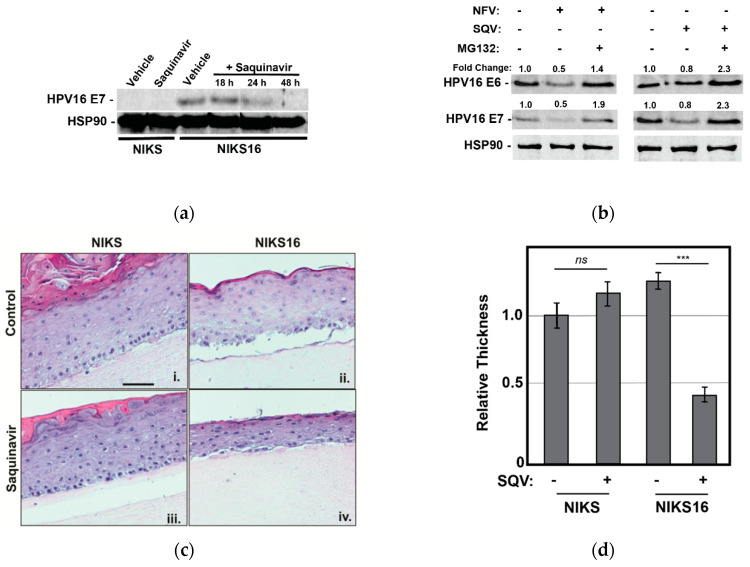
Saquinavir causes loss of E6 and E7 expression in HPV16-positive NIKS16 cells and causes atrophy in NIKS16 3-D organotypic raft cultures. (**a**) NIKS and NIKS16 cells were treated with 5 µM SQV for 18–48 h, with cell lysates harvested and probed for HPV E7 and HSP90 (control) as for Figure 1a. (**b**) NIKS16 cells were treated with either 10 µM SQV or 10 µM NFV for 48 h before adding 5 µM MG132 for 3 h prior to harvesting cell lysates. E6 and E7 levels were detected and measured using quantitative immunoblot. Relative values were normalized to the vehicle control to show fold change. Data are representative of three biological replicates. (**c**) Organotypic raft culture using NIKS and NIKS16 cells in the presence or absence of SQV. Parental NIKS and NIKS16 cells were rafted on collagen for 7 days prior to treatment with 5 µM SQV or vehicle control with drug refreshed every 48 h for 8 additional days prior to fixation, embedding in paraffin, and H&E staining. Scale bar represents 50 µm. (**d**) The thickness of each raft (*n* = 3) was measured using NIH ImageJ, with values normalized to the thickness of the untreated NIKS control. Error bars represent the standard deviation of the mean for five slices derived from each sample, for three technical experimental replicates. ns = not significant and *** = *p* ≤ 0.0005 based on a Student’s two-tailed *t*-test.

We first confirmed that 5 µM SQV reduced HPV E7 expression over time in NIKS16 cells grown in 2-D culture prior to rafting, observing precipitous loss to E7 levels over a 48 h time course, as expected (Figure 4a). We also confirmed that MG132 treatment reversed the effects of both NFV and SQV on E6 and E7 depletion in NIKS16 cells (Figure 4b), similar to what we had observed for CaSki cells (Figure 3c), indicating that these drugs affect oncoprotein levels in both cell types using a similar mechanism. 

We subsequently rafted NIKS or NIK16 cells for 7 days prior to adding 5 μM SQV or vehicle control (DMSO) for an additional 8 days with the drug refreshed every 2 days, with SQV chosen for these experiments because NFV was observed to cause relatively greater non-specific cytotoxicity in long-term cultures. SQV-treated NIKS16 rafts exhibited an atrophic morphology (reduced epithelial thickness) relative to either vehicle-treated NIKS16 cells or NIKS grown in the presence or absence of SQV treatment (Figure 4c,d). These results revealed that SQV can affect the growth kinetics of HPV16-positive cells even in a pre-cancer state.

**Figure 5:**
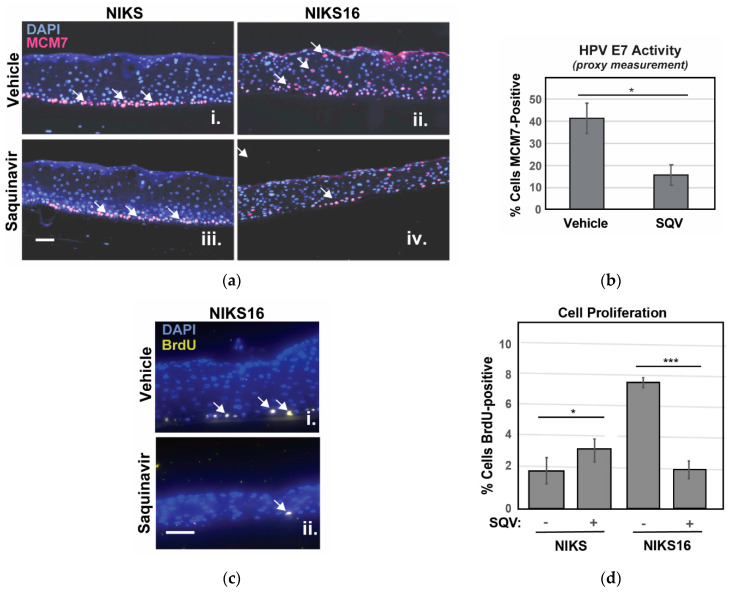
SQV selectively slows growth of NIKS16 rafts. (**a**) NIKS and NIKS16 rafts were sectioned and stained with DAPI (blue) to visualize nuclei and E7-specific marker MCM7 (red, highlighted using white arrows) using anti-MCM7 antiserum. Size bar represents 25 µm. (**b**) Percentage of NIKS16 cells that were MCM7-positive with or without SQV treatment. Error bars represent the standard deviation from the mean for three technical replicates (>100 cells per section). (**c**) Detection of BrdU (yellow, highlighted using white arrows) to measure cell proliferation in NIKS16 cells in the presence or absence of SQV. Nuclei were visualized using DAPI (blue) and BrdU detected using anti-BrdU antiserum. Size bar represents 25 µm. (**d**) Quantification of nuclei positive for BrdU. Error bars represent the standard deviation from the mean for five technical replicates (>70 cells per section); * = *p* ≤ 0.05, *** = *p* ≤ 0.0005 based on a Student’s two-tailed *t*-test.

We were unable to detect E6 and E7 by immunohistochemistry so, to confirm suppression of E7 in NIKS16 epithelia, we instead stained sectioned rafts for MCM7 protein, encoded by an E2F-responsive gene normally expressed only in basal cells, but also expressed in suprabasal cells in the presence of E7 [54]. As anticipated, MCM7-positive cells were observed in the suprabasal layer of NIKS16 rafts but not in the HPV-negative NIKS (Figure 5a). After SQV treatment, fewer MCM7-positive cells were observed in NIKS16 rafts, consistent with a loss of E7 activity (Figure 5b). BrdU pulse assay was also used to measure cell proliferation, confirming slower growth of NIKS16 cells treated with SQV compared to otherwise identical rafts treated with vehicle control, with no negative effects of SQV on HPV-negative NIKS rafts (Figure 5c,d). These results indicated that SQV was also able to inactivate E7 in the context of a stratified epithelium and, interestingly, could cause significant reductions of overall HPV16-positive cell growth over time.

**Figure 6:**
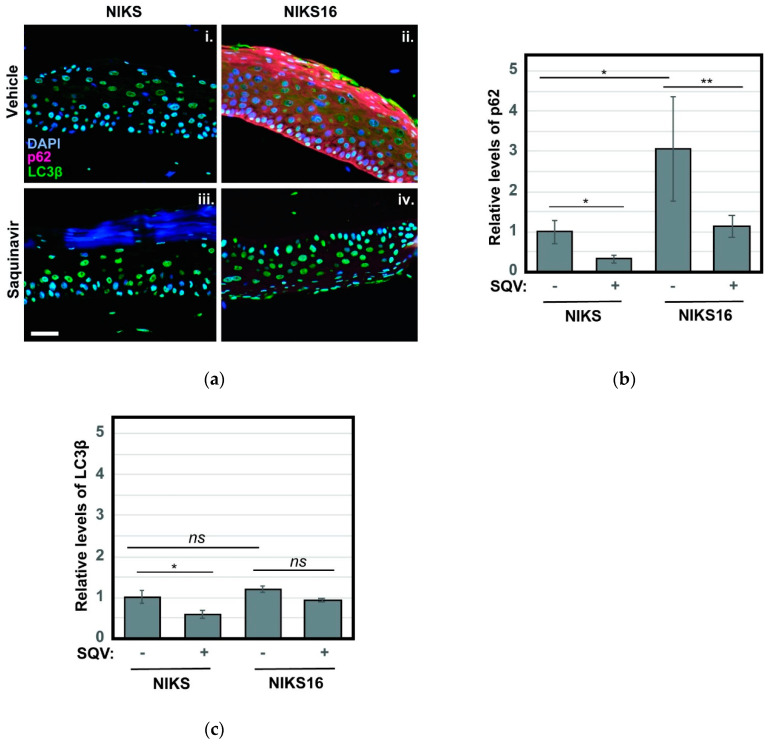
SQV reduces HPV16-associated increases to markers of cellular autophagy. (**a**) NIKS and NIKS16 rafts were sectioned and stained using DAPI (blue) and for the autophagy markers p62 (red) and LC3β using anti-p62 and anti- LC3β antisera, respectively. Size bar represents 100 µm. (**b**,**c**) Quantification of p62 (**b**) and LC3β (**c**) levels for each condition. For bar graphs, error bars represent the standard deviation from the mean for >3 technical replicates; ns = not significant; * = *p* ≤ 0.05, ** = *p* ≤ 0.005 based on a Student’s two-tailed *t*-test.

Based on recent reports that inhibition of autophagy is crucial to HPV16-driven cancer development [37,55,56], we also stained NIKS and NIKS16 rafts treated with or without SQV to detect the autophagy markers p62 and LC3β. Autophagy (“self-eating”) is a mechanism by which metabolically stressed cells clear cytosolic aggregates or damaged organelles through their engulfment in ER-derived autophagosomes that subsequently fuse with degradative lysosomes [57,58]. HPV16 E6 and E7 were recently shown to interfere with this pathway, preventing the final step of autophagosome fusion with lysosomes [59], and we had previously established elevated levels of p62 and LC3β as markers of HPV16-driven pre-cancer [56]. Consistent with HPV16 inhibiting the last stage of autophagy, we observed a striking ~3-fold increase to the levels of p62, a well-known target of autophagy-mediated degradation used to monitor late autophagosome activity, in NIKS16 rafts when compared to HPV-negative NIKS rafts (Figure 6a, compare panels i and ii). Remarkably, SQV treatment reduced p62 levels in NIKS16 cells to wild-type (NIKS) levels (Figure 6b), consistent with the notion that SQV treatment is able to reverse HPV16′s capacity to inhibit autophagy. LC3β levels were not as elevated in NIKS16 cells and SQV caused mild reductions of this marker in both NIKS and NIKS16 rafts (Figure 6a with quantification in Figure 6c).

Taken together, these results using NIKS16 cells demonstrate that SQV can reduce the growth of HPV16-positive cells in the context of a 3-D epithelium, affecting HPV16-associated cancer phenotypes. Importantly, these effects appear targeted, with SQV having little to no impact on the growth of HPV-negative raft cultures. 

## 3. Discussion

E6 and E7 are the driving forces for HPV-associated carcinogenesis and their targeted inactivation could provide the basis for an effective anti-HPV-driven cancer therapy. In this study, we report the novel finding that at least four currently FDA-approved protease inhibitors (LPV, RTV, SQV, and NFV) markedly reduce HPV16 E6 and E7 levels in two independent cell culture models: HPV16+ CaSki cervical cancer cells and immortalized human foreskin keratinocytes harboring HPV16 extrachromosomal genomes (NIKS16). A subset of these drugs (LPV, RTV, and SQV) caused cell growth arrest in CaSki cancer cells (Figure 2). Moreover, treatment of NIKS16 raft cultures with SQV caused epithelial atrophy and reversal of HPV-triggered increases to markers of cellular autophagy (most notably, p62), with no apparent negative growth effects on HPV-negative NIKS raft cultures (Figure 4 and Figure 5). Based on these selective, cancer-relevant properties, we propose that HIV protease inhibitors be studied more extensively as potential agents for targeted treatment or prevention of HPV-associated cancers. 

We were surprised to find that the two protease inhibitors we studied for mechanism, NFV and SQV, induced loss of E6 and E7 post-transcriptionally through a pathway at least partially linked to the proteasome (Figure 3c and Figure 4a,b). One interpretation of these findings is that NFV and SQV induce selective proteasome-mediated degradation of the two viral oncoproteins, which would be remarkable given that protease inhibitors were previously posited to cause inhibition of host proteasomes—an activity that, if global, would be predicted to have the opposite effect on E6 and E7 [49]. Indeed, a prior study showed SQV treatment of HPV-positive HeLa cells to increase overall levels of ubiquitinated proteins at the whole cell level [35]), albeit at levels of drug much higher than those tested in our study (>60 µM). Also unclear is what is common about E6 and E7 that would make these two proteins similarly subjected to the effects of these drugs. E6 and E7 lack amino acid sequence similarity and have little overt structural similarity, with the exception of possessing zinc finger motifs [6,7,60].

That said, both E6 and E7 have been shown to be targets for proteasome-mediated degradation in prior studies [61,62]. E6 recruits the ubiquitin ligase E6AP to target p53 for proteasome-mediated degradation, and a recent paper argued that E6 can be similarly targeted by E6AP for degradation [63]. E7, which recruits the ZER1/CUL2-RING ubiquitin ligase complex to promote degradation of pRb [64], has also been reported to be targeted itself for proteasome-mediated degradation by the SOCS1 adaptor complex [64] and/or a SCF (Skp-Cullin-F box) ubiquitin ligase complex containing Cullin 1 (Cul1) and Skp2 [65,66]. HPV16 E7′s core stability element has been mapped to its N-terminal domain and is regulated in an interesting fashion, with a first ubiquitin linearly conjugated to the N-terminus followed secondary ubiquitination of internal lysine residues [61]. Both E6 and E7 have also been found to be targets for the deubiquitinases (DUBs) USP11 [67] and USP15 [68], respectively, and a recent, directed screen of components of the ubiquitin-proteasome system using a split luciferase complementation assay found E6 and E7 associated with factors involved in both ubiquitination and de-ubiquitination [69]. These findings lead us to an intriguing alternative hypothesis, that protease inhibitors reduce the levels/activity of the DUBs that target these viral oncoproteins, thereby causing increased ubiquitination of E6 and E7, and consequently increased degradation. However, although E7 was sensitive to both NFV and MG132, unlike for p53 we did not observe an additive effect when both drugs were combined (Figure 3D), a result inconsistent with this particular hypothesis. Additional studies will clearly be needed to fully understand how ubiquitination or deubiquitination (and potentially translation regulation) underpin the effects of protease inhibitors on E6 and E7, with E6AP being an unlikely determinant considering its established role in degrading p53 [70]. 

Our second important observation was that SQV suppresses growth of immortalized NIKS16 cells in 3-D raft cultures with little to no observed effects on HPV-negative NIKS rafts (Figure 4c,d). These selective properties demonstrate targeted drug activity in a relevant model for HPV-driven precancer. The results also suggest that NIKS16 rafts are addicted to HPV16 oncoprotein expression, potentially due to HPV16′s inhibition of autophagy even in this scenario (Figure 6). That SQV both suppresses NIKS16 raft growth and blocks HPV16-driven increases to markers of autophagy are encouraging results in the context of exploring protease inhibitors as potential agents for suppressing HPV16-driven cancer phenotypes.

It is important to reiterate that HIV protease inhibitors are already known to exhibit general anticancer properties even for HPV-negative cancers. NFV has been shown to modulate PI3K/mTOR signaling in a variety of cancer cell types resulting in cell death; this is thought to be triggered by NFV-mediated induction of ER stress due to general inhibition of host proteasomes [71,72,73,74,75,76]. Alternatively, protease inhibitors can exert anticancer effects through inhibition of matrix metalloproteases [32,76,77,78]. In the context of HPV cancers, Hampson and colleagues have shown that a vaginal pessary delivering Lopimune (LPV and RTV) was able to treat high-grade HPV-positive cervical intraepithelial neoplasia (CIN) in women, with a >80% positive response rate [36]. An additional cell culture study from this group demonstrated that cervical cancer cells increase p53 levels in response to lopinavir [34], results consistent with our data (Figure 2b and Figure 3a) and potentially explained by our observations that protease inhibitors target and deplete E6 from HPV16-positive cell types (Figure 1, Figure 2b and Figure 3a). 

In this context, protease inhibitors might also be evaluated as potential chemosensitizers for combination anticancer therapies. For example, the antiviral drug cidofovir, an inhibitor of herpesvirus DNA polymerase, dampens E6 and E7 expression at the transcriptional level [79] and was shown to cause regression of cervical cancer tumors when administered in combination with cisplatin and radiotherapy in a Phase I clinical trial [80]. Similarly, JQ1, a small molecule inhibitor of bromodomain or extraterminal domain (BET) protein BRD4, downregulates *E6* and *E7* transcription [81] and was shown to render otherwise chemo-resistant cervical cancer cells vulnerable to cisplatin in vitro [82]. Relatedly, prior work from our labs demonstrated that an autophagic inducer, PI3K/mTOR inhibitor BEZ235, slows progression of HPV-positive anal cancers in a HPV16 E6/E7 mouse model [83], so protease inhibitors might also be studied in combination with autophagy inducers to determine if E6/E7 downregulation potentiates drugs developed to suppress this cancer-linked pathway.

Finally, although protease inhibitors have been used by PLWH as part of HAART for many years without substantial benefit in preventing HPV-driven cancers [26,27], in these instances, drugs were taken orally and it is possible that it is difficult to achieve sufficient therapeutic concentrations in peripheral infected epithelial tissues. Thus, continual, high dose administration of protease inhibitors may be critical to their anti-HPV cancer therapeutic potential. Instead, repeated topical local application might be considered, as was pursued in the recent CIN trial [36].

## 4. Materials and Methods 

### 4.1. Cell Lines and Drugs

CaSki (HPV16-positive human cervical cancer cells), NIKS (normal immortalized keratinocytes), NIKS16 (NIKS containing HPV16 episomes), 3T3 (mouse fibroblast cells) have been previously described [84,85]. CaSki cells were grown in RPMI 1630 media supplemented with 10% fetal bovine serum. 3T3 cells were grown in DMEM media supplemented with 10% fetal bovine serum. NIKS and NIKS16 were cultured and grown in raft culture as previously described [84,85]. HIV-1 protease inhibitors atazanavir, indinavir, lopinavir, nelfinavir, ritonavir, and saquinavir were obtained from the NIH AIDS Reagent Program, Division of AIDS, NIAID, NIH (Germantown, MD, USA). 

### 4.2. Immunoblots

Cells were lysed in HNTG buffer (50 mM HEPES pH 7.5, 150 mM NaCl, 0.1% Triton X-100, 1 mM EGTA, 10% Glycerol) supplemented with protease inhibitor cocktail (Roche) and 1 mM PMSF. Whole-cell lysates were resolved on a 12% SDS-PAGE gel and transferred to nitrocellulose membranes. Subsequently, membranes were blocked for 1 h at room temperature in 5% skim milk-TBST (TBST is 0.1% Tween 20-TBS). A primary antibody mixture containing 1:2000 dilution of anti-E6 (GTX132686, Genetex), 1:2000 dilution of anti-E7 (GTX133411, Genetex), 1:300 dilution of anti-p53 (sc-126, Santa Cruz), 1:300 dilution of anti-pRb (sc-102, Santa Cruz), 1:1000 anti-p24^Gag^ (183-H12-5C, NIH AIDS Reagent Program), or HSP90 (sc-7947, Santa Cruz) antisera were added in blocking buffer overnight at 4 °C. After washing with TBST, blots were incubated with 1:10,000 dilution of secondary antibodies in blocking buffer (IRDye 680LT goat anti-mouse IgG or IRDye 800CW goat anti-rabbit IgG secondary antibodies, LI-COR) for 1 h at room temperature. Blots were washed with TBST and visualized using an LI-COR Odyssey infrared imaging system. Band intensity was analyzed using the Image Studio program. Full versions of the immunoblots featured in the paper are included as Figure S1.

### 4.3. Cell Viability Assay

Cell viability was determined by CellTiter-Glo Luminescent Cell Viability Assay (Promega). Briefly, 10^4^ cells were plated in 96-well plates and cultured in the presence of HIV-1 protease inhibitors. After 3 days, CellTiter-Glo Reagent was added to the cell culture medium as per the manufacturer’s instructions, mixed on a shaker for 2 min, and incubated at room temperature for 10 min to stabilize the luminescent signal. The plates were read using a VICTOR Multilabel Plate Reader (Perkin Elmer). 

### 4.4. Cell Cycle Analysis 

Equivalent numbers of cells incubated with HIV-1 protease inhibitors were collected and centrifuged for 10 min at 300× *g*. After washing with PBS, cells were resuspended in 0.5 mL PBS and then transferred to 4.5 mL of 70% cold ethanol overnight at −20 °C to fix the cells. Subsequently, cells were centrifuged, washed with PBS, and suspended in DAPI/0.1% Triton staining solution overnight at 4 °C. Flow cytometry data were acquired using the Attune Map and analyzed using ModFit (Verity Software House).

### 4.5. Raft Culture

To produce organotypic rafts, NIKS or NIKS16 cells were cultured as previously described [84,85]. Seven days after the collagen dermal equivalent was lifted to the air-liquid interface, either 5μM Saquinavir or DMSO was added to the culture medium for 8 days with the drug refreshed every two days. For cell proliferation analysis, 10μM bromodeoxyuridine (BrdU) was added to the medium 8 h prior to harvesting the rafts. After the rafts were harvested, they were embedded in paraffin and cut into 5 μm sections.

### 4.6. Immunofluorescence Imaging

Immunostaining of raft sections was performed as previously described [78]. To detect MCM7, MCM7 antiserum (sc-65469, Santa Cruz, Dallas, TX) was used at a 1:200 dilution. For BrdU detection, BrdU antiserum (NA-61, Millipore, Burlington, MA) was used at a 1:50 dilution. Staining for p62 and LC3β was carried out using anti-p62 (Abcam) and anti-LC3β (Santa Cruz) antisera, respectively, as previously described [83].

## 5. Conclusions

High-risk strains of HPV are known to be causal agents for oropharyngeal cancers and anogenital cancers, including cervical cancer, of which almost all cases are HPV-positive. In cervical cancers, E6 and E7 are always expressed and their cessation of expression causes regression of cancers. In this study, we found that some but not all FDA-approved HIV protease inhibitors decreased the levels of E6 and E7 expression in two independent HPV16-positive cell culture models: CaSki cervical cancer cells and NIKS16 3-D organotypic rafts. Protease inhibitor effects on E6 and E7 were correlated with loss of cell growth in both systems and it was notable that saquinavir’s effects on NIKS16 rafts were selective, causing epithelial atrophy and reversal of HPV-induced upregulation of markers of autophagy with little to no apparent effects on the growth of HPV-negative raft cultures. These findings are encouraging in the context of repurposing HIV protease inhibitors for targeted treatment of HPV-positive cancers. Subsequent studies will be targeted at further defining the mechanism(s) of drug selectivity both in vitro and using in vivo models. 

## Data Availability

Not applicable.

## Supplementary Materials

The following are available online at https://www.mdpi.com/2072-6694/13/5/949/s1, Figure S1: Full versions of the immunoblots featured in the paper.
