# Supplementary material for: HIV-1 Protease Inhibitors Slow HPV16-Driven Cell Proliferation through Targeted Depletion of Viral E6 and E7 Oncoproteins
Source: Cancers (Basel). 2021 Feb 24;13(5):949. doi: 10.3390/cancers13050949 (PMC7956332; doi:10.3390/cancers13050949)

FIGURE 1- Original Blots

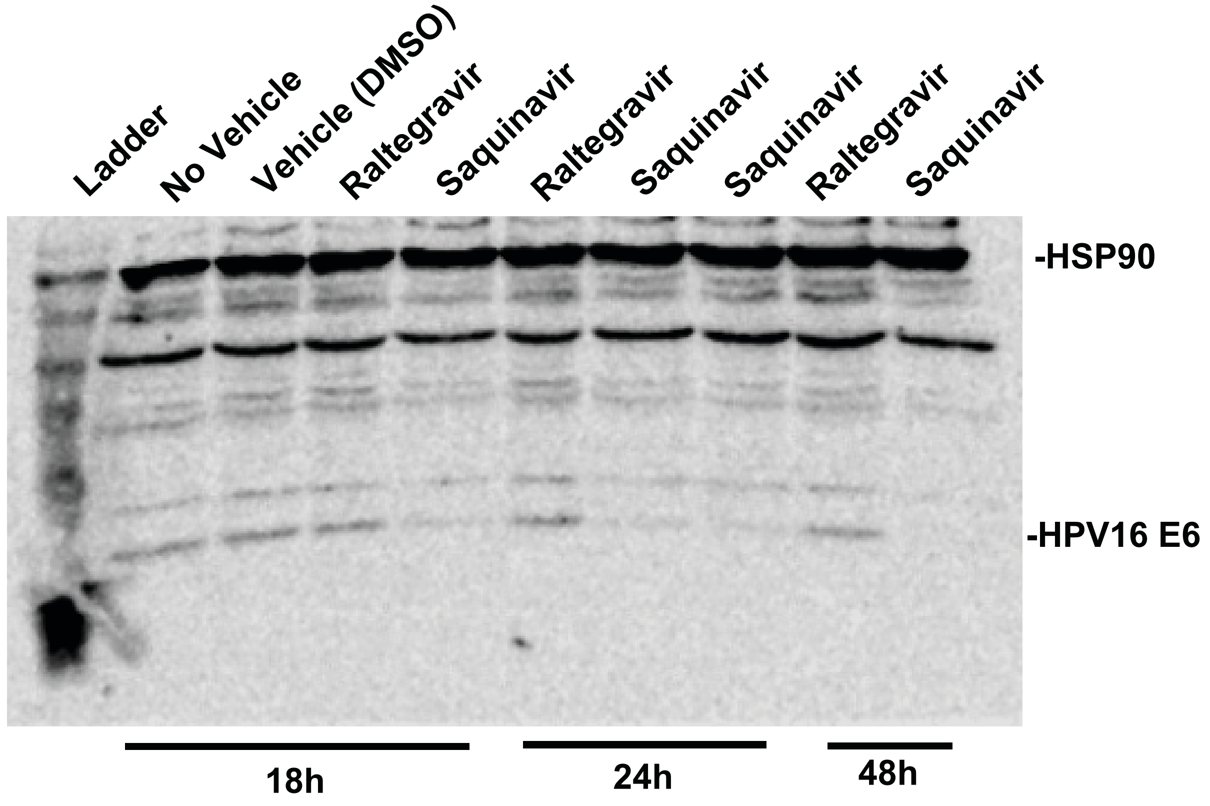

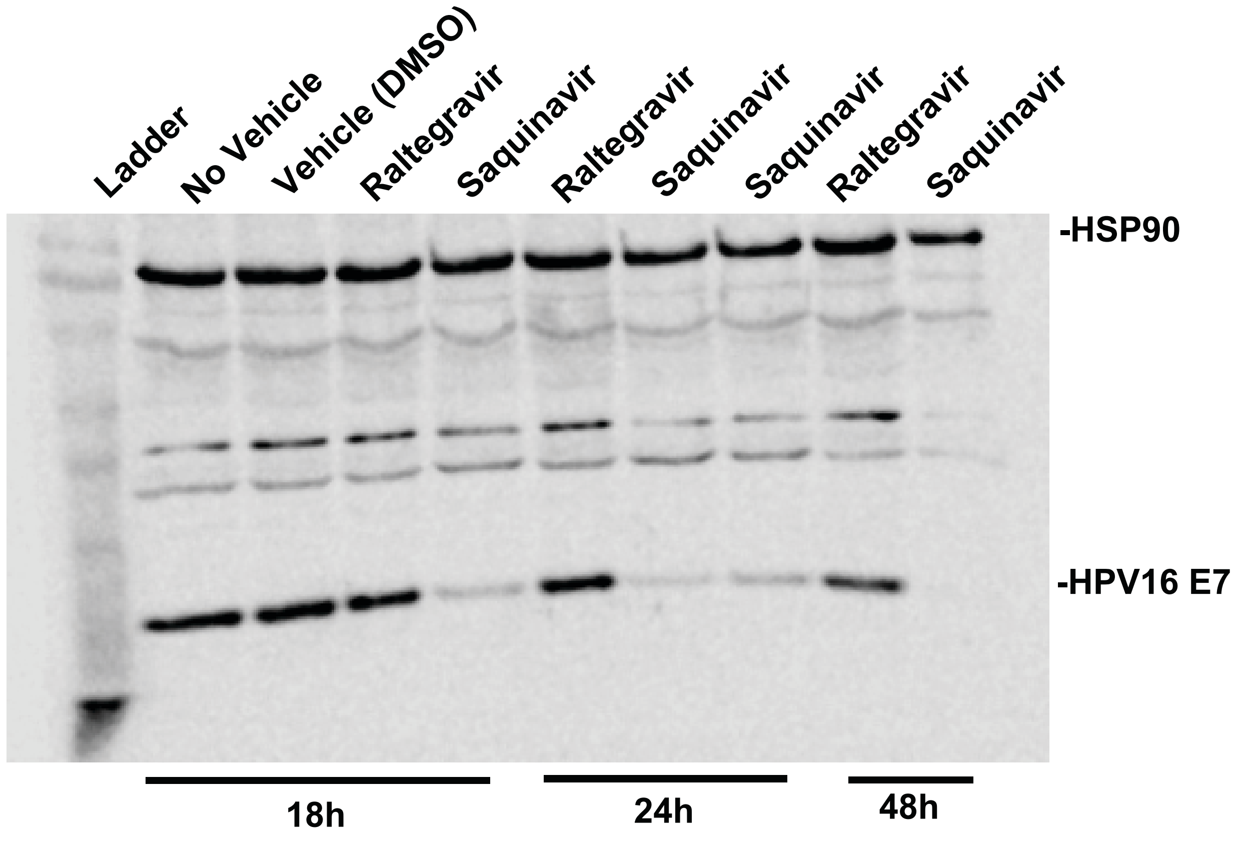

FIGURE 2A- Original Blot

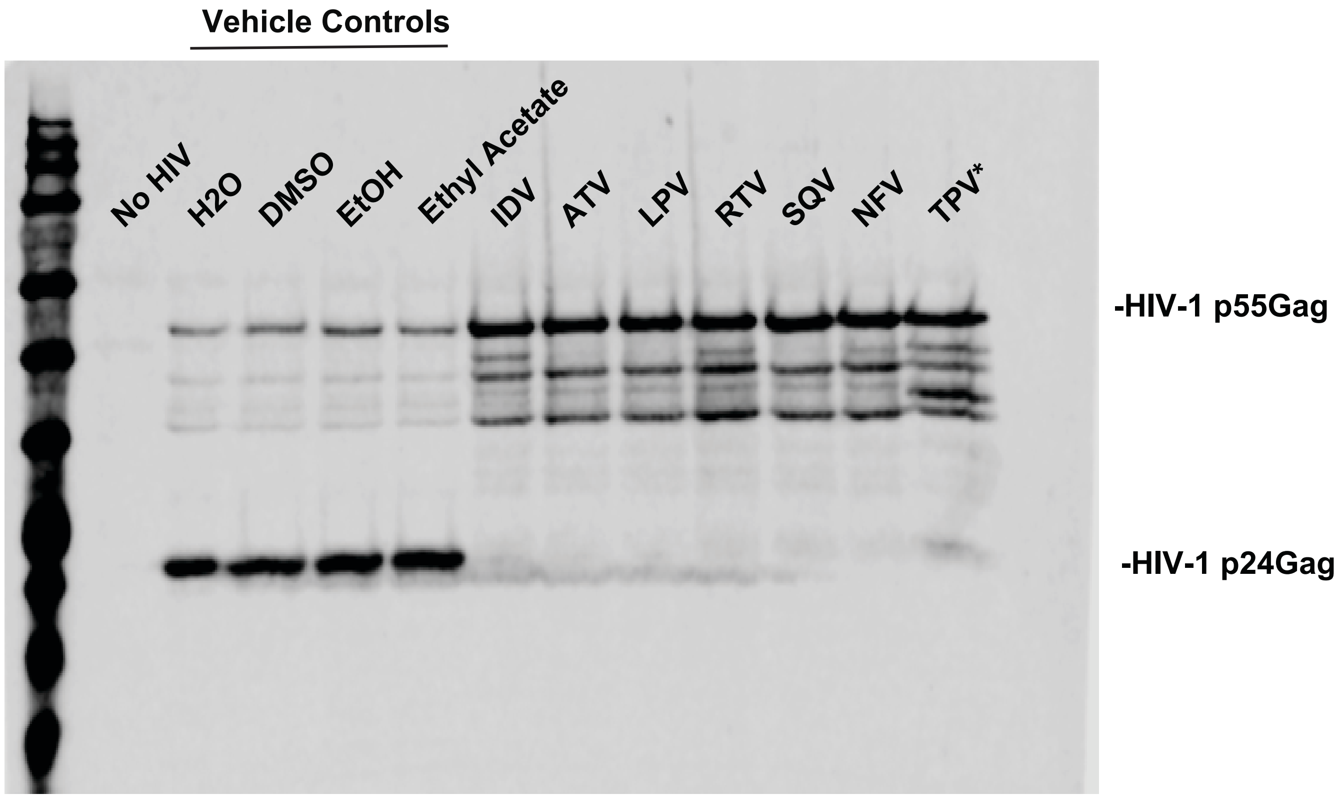

*Note- Tipranavir (TPV) was tested in this experiment but excluded from Figure 2A because it was not included in the cell viability and cell cycle analyses.

FIGURE 2B- Original Blots

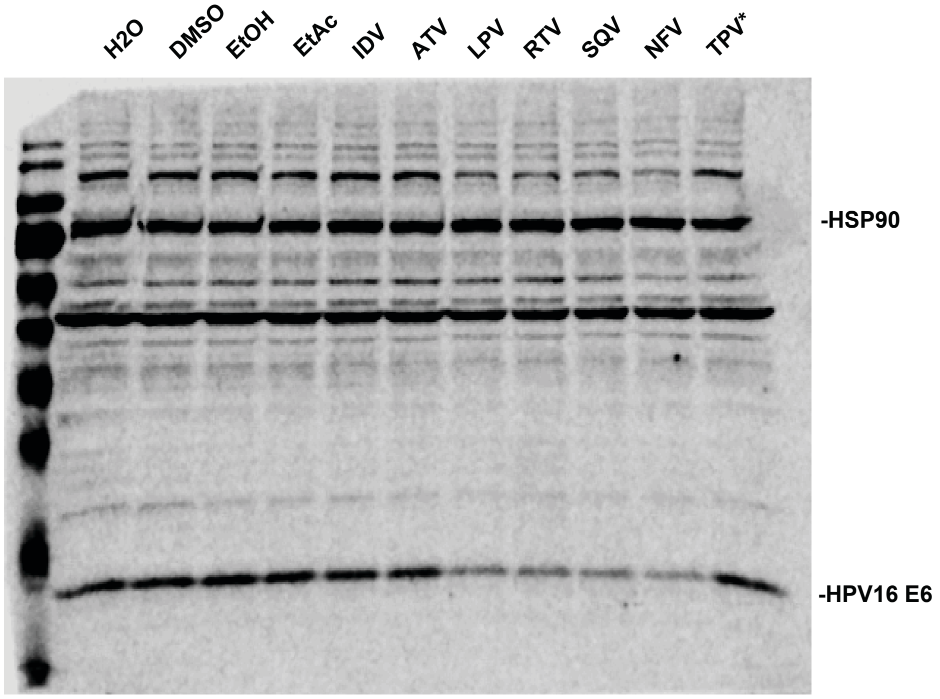

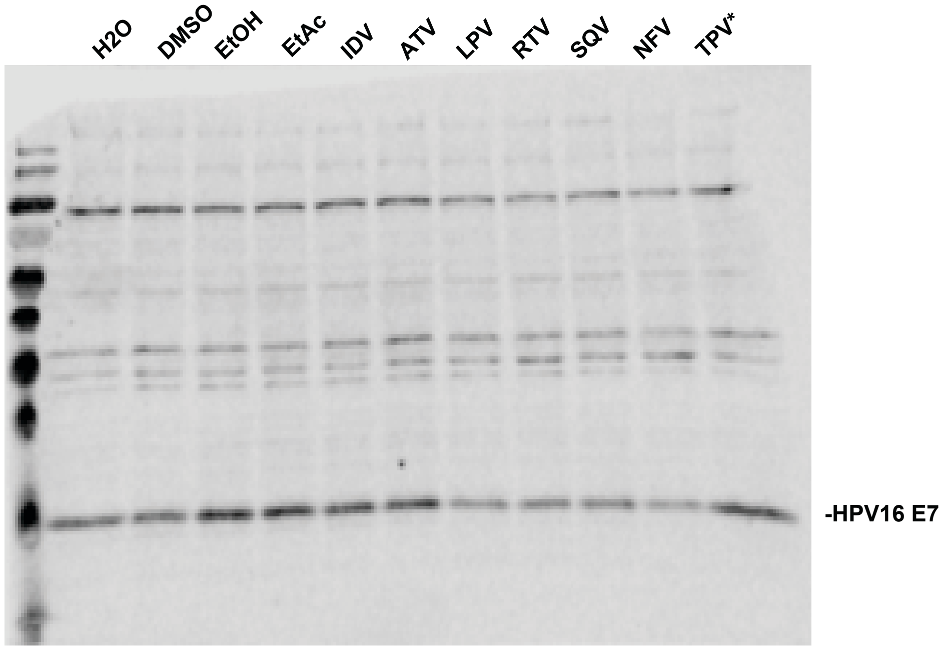

*Note- Tipranavir (TPV) was tested in this experiment but excluded from Figure 2B because it was not included in the cell viability and cell cycle analyses.

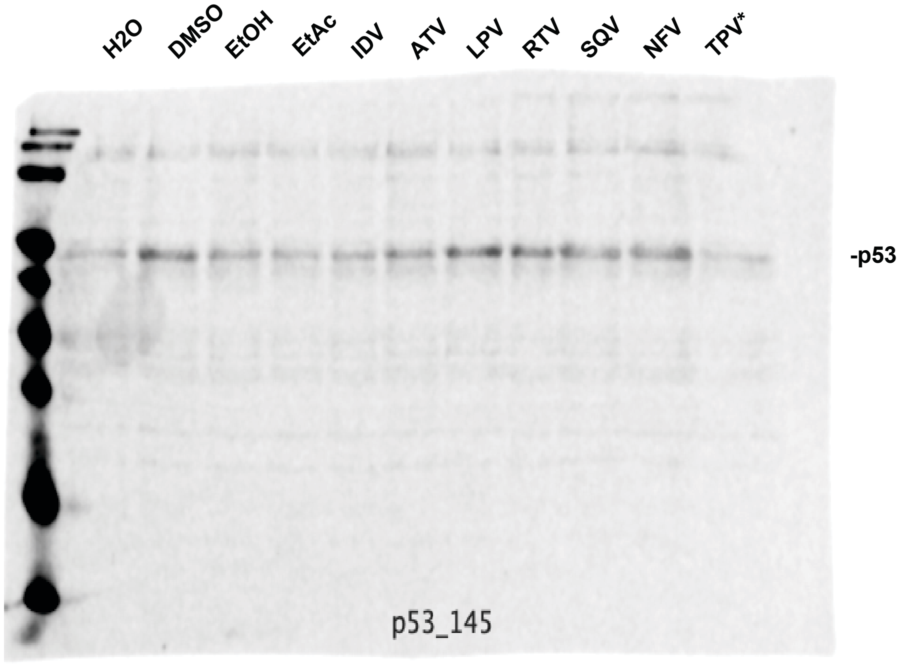

FIGURE 3A- Original Blots

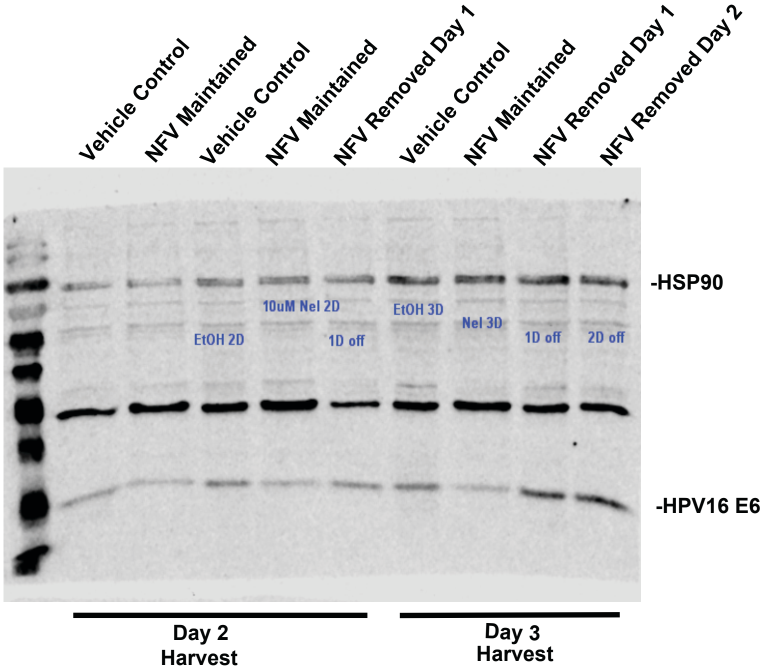

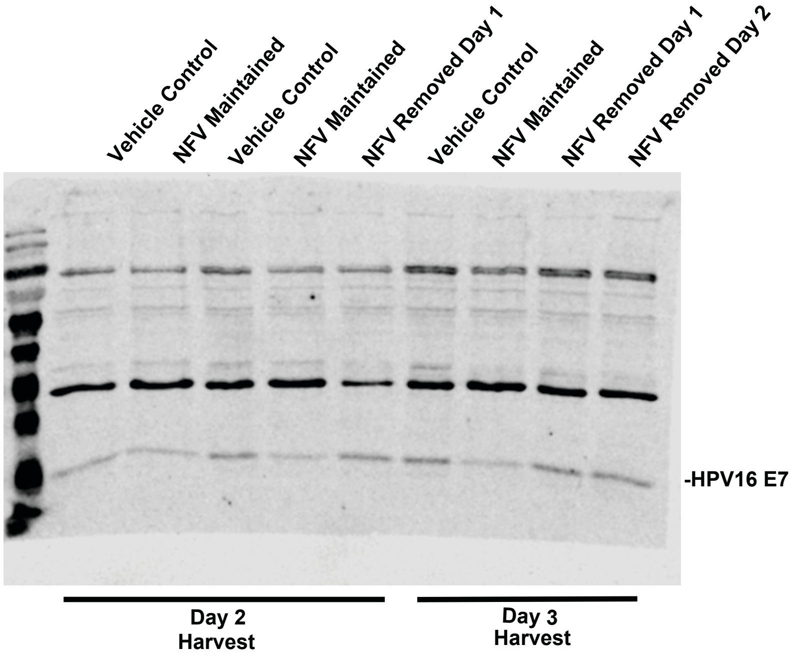

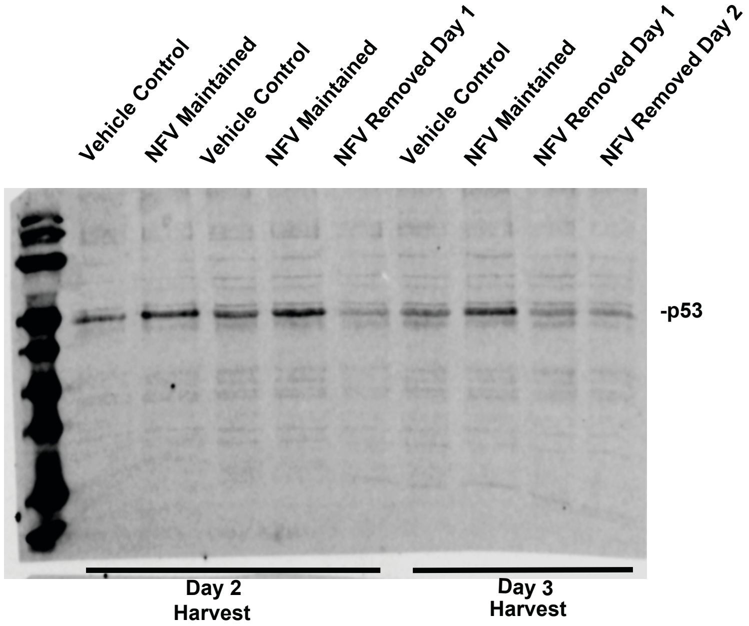

FIGURE 3C- Original Blots

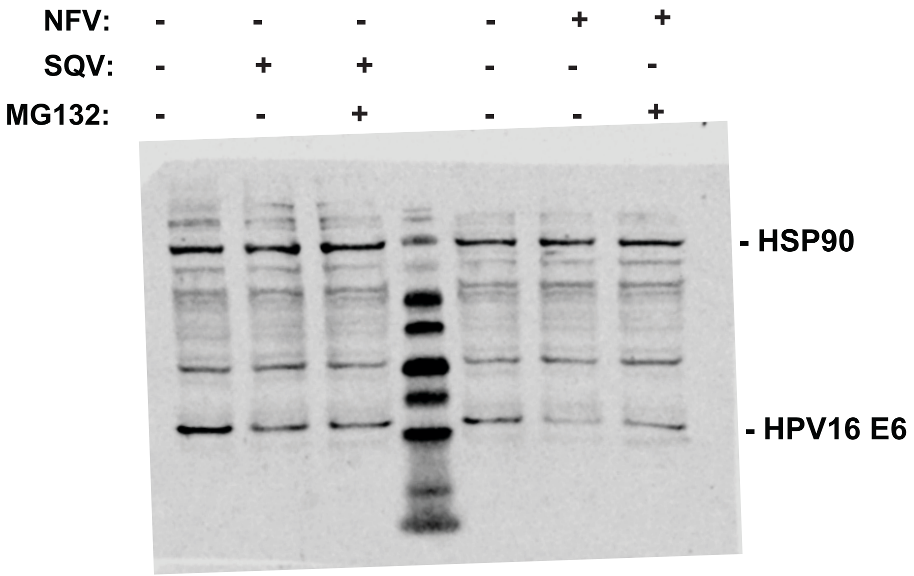

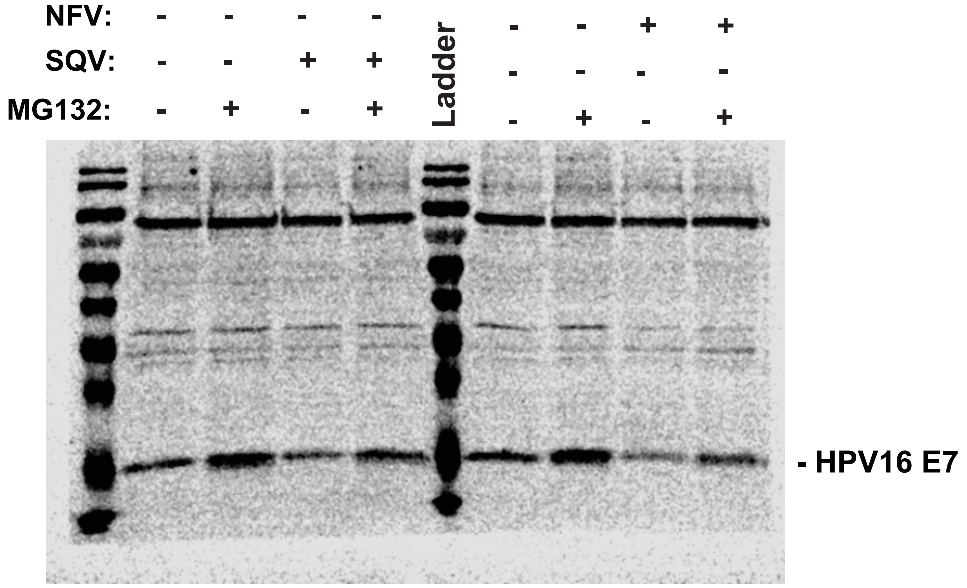

FIGURE 3D- Original Blots

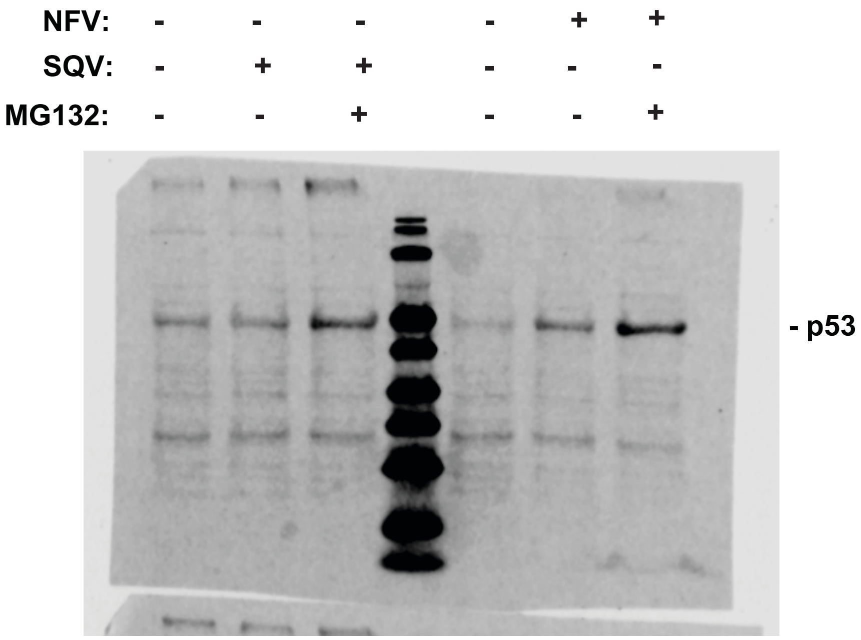

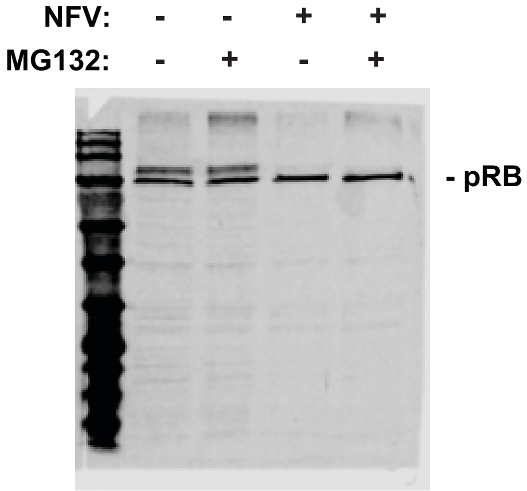

FIGURE 4A- Original Blots

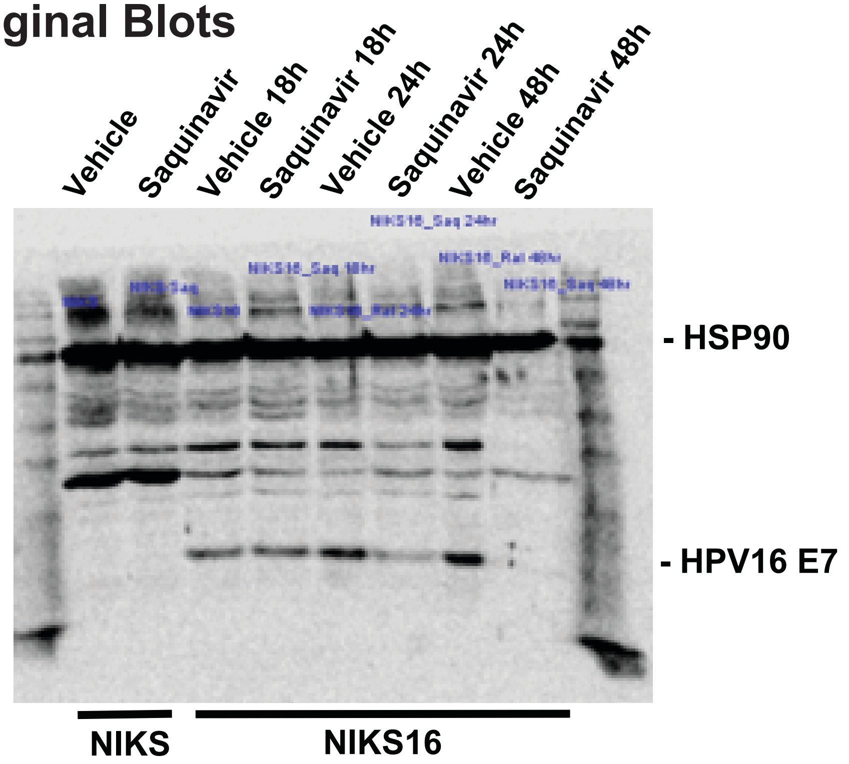

FIGURE 4B- Original Blots

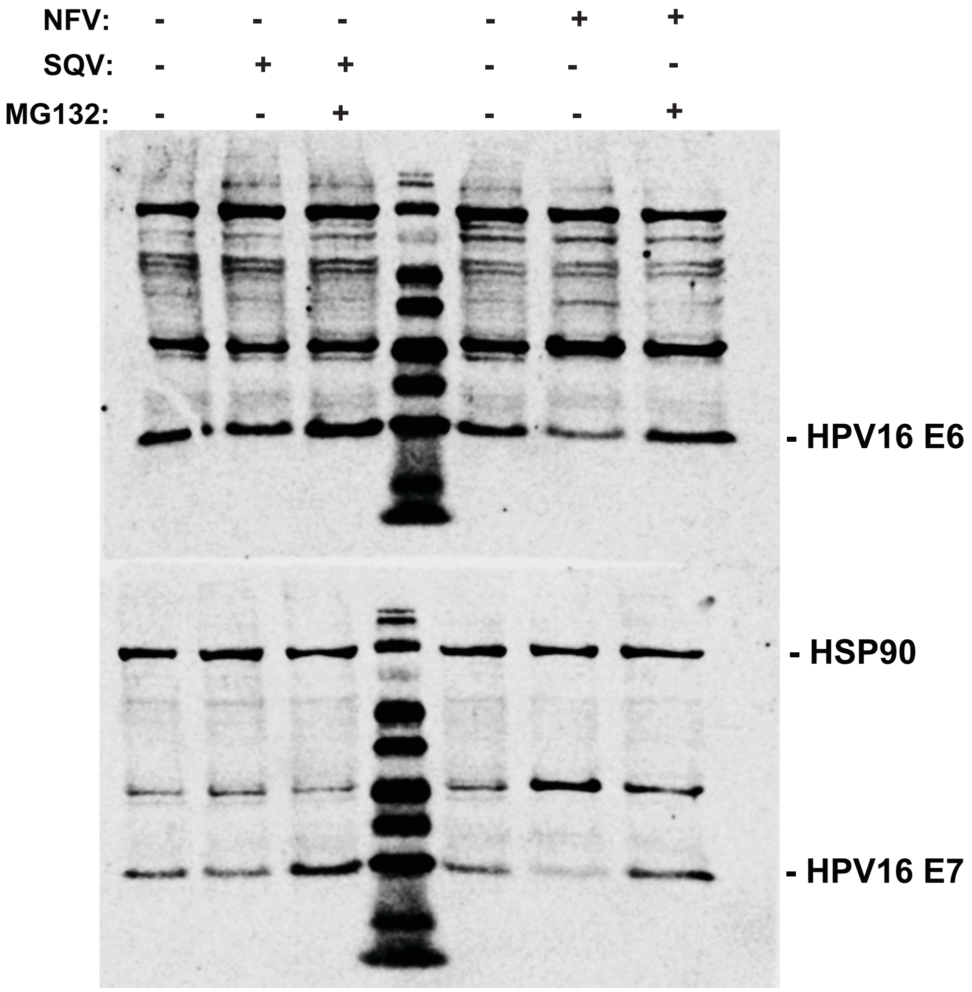

Supplement: Supplementary file 1 [file cancers-13-00949-s001.pdf]
